# Emergent expression of fitness-conferring genes by phenotypic selection

**DOI:** 10.1093/pnasnexus/pgac069

**Published:** 2022-06-10

**Authors:** Marta Ciechonska, Marc Sturrock, Alice Grob, Gerald Larrouy-Maumus, Vahid Shahrezaei, Mark Isalan

**Affiliations:** Department of Life Sciences, Imperial College London, London SW7 2AZ, UK; Department of Physiology, Royal College of Surgeons in Ireland, Dublin D02 YN77, Ireland; Department of Life Sciences, Imperial College London, London SW7 2AZ, UK; Department of Life Sciences, Imperial College London, London SW7 2AZ, UK; Department of Mathematics, Imperial College London, London SW7 2AZ, UK; Department of Life Sciences, Imperial College London, London SW7 2AZ, UK

## Abstract

Genotypic and phenotypic adaptation is the consequence of ongoing natural selection in populations and is key to predicting and preventing drug resistance. Whereas classic antibiotic persistence is all-or-nothing, here we demonstrate that an antibiotic resistance gene displays linear dose-responsive selection for increased expression in proportion to rising antibiotic concentration in growing *Escherichia coli* populations. Furthermore, we report the potentially wide-spread nature of this form of emergent gene expression (EGE) by instantaneous phenotypic selection process under bactericidal and bacteriostatic antibiotic treatment, as well as an amino acid synthesis pathway enzyme under a range of auxotrophic conditions. We propose an analogy to Ohm’s law in electricity (V = IR), where selection pressure acts similarly to voltage (V), gene expression to current (I), and resistance (R) to cellular machinery constraints and costs. Lastly, mathematical modeling using agent-based models of stochastic gene expression in growing populations and Bayesian model selection reveal that the EGE mechanism requires variability in gene expression within an isogenic population, and a cellular “memory” from positive feedbacks between growth and expression of any fitness-conferring gene. Finally, we discuss the connection of the observed phenomenon to a previously described general fluctuation–response relationship in biology.

Significance Statement Phenotypic selection is a potential mechanism for cells to respond to selection pressures, such as antibiotic or cancer drug exposure. Understanding mechanisms rooted in transient gene expression variation will help predict and prevent development of drug resistance. We use synthetic biology approaches to demonstrate that antibiotic resistance genes display linear dose-responsive upregulation in proportion to antibiotic concentration—which we refer to as emergent gene expression (EGE). Using mathematical modeling, we show the observed EGE is due to phenotypic selection which requires noisy gene expression, and some level of inheritance or memory of expression of any fitness-conferring gene after cell division.

## Introduction

Isogenic microbial populations within the same environment were often assumed to be physiologically uniform. However, contrary to this belief, populations are known to exhibit phenotypic variability such as expressing genes at variable levels due to stochastic gene expression (1,2). In the absence of any genetic differences in the cell population, this “noisy” gene expression can be attributed to a number of different intrinsic or extrinsic sources including the innate stochasticity of biochemical reactions dependent on a small number of molecules, transcriptional and/or translational bursting and differences in cell cycle progression (3). The differences in gene expression among a clonal population can provide a survival strategy in fluctuating environments and is known as bet-hedging (4). Indeed, relying on preexisting phenotypic variability could be a better strategy than sensing and responding to environmental fluctuations (5, 6). An interesting example of this strategy has been demonstrated by Bishop et al. (7), who compared the survival of WT *Saccharomyces cerevisiae* with a mutant strain with an increased sensitivity to perturbations, but which displayed wide phenotypic heterogeneity. It was found that the mutants initially exhibited sensitivity to multiple perturbations, including exposure to nickel, copper, and alkaline pH. However, the broad heterogeneity meant that certain individuals within the mutant population displayed a higher level of resistance, enabling population survival. It should be noted though, that fitness can increase continuously (following a nonsaturating power–law function) in populations that face the same initial stress (glucose limitation) throughout its evolutionary history, and thus do not experience frequent changes in environmental conditions (8).

Bet-hedging is commonly defined as a risk-spreading strategy in which a population, through stochastic switching, is able to form subpopulations of distinct phenotypes (9). Although this reduces the mean fitness of the population, the likelihood of species survival during environmental catastrophes is greatly enhanced (10). Cells among the population that express advantageous adaptive machinery are better equipped to survive random environmental fluctuations, however, the production of such machinery comes at a fitness cost under favorable conditions (9). Bet-hedging is often described in bistable populations, where cells are able to stochastically switch between two phenotypes, independent of any endogenous signaling pathways, as suggested for bacterial persistence (11,12). This is termed an “all-or-nothing response,” where a specific gene or group of genes are either expressed or not expressed. Examples of bet hedging in bistable populations are seen in the formation of fimbriae in *Escherichia coli* (13), sporulation and biofilm production in *Bacillus subtilis* (14), and also competence in *B. subtilis* (15,16). Both experimental evolution studies in Pseudomonas bacteria and in silico evolution studies have shown that a bistable system can emerge as a bet-hedging mechanism in fluctuating environments (17,18).

While natural selection is traditionally thought to act on genotypes, in a bet-hedging scenario, the fit state can be selected in the favorable environment due to its fitness advantage. This phenomenon is nicely demonstrated by Kashiwagi et al. (19), who used a synthetic genetic toggle switch in *E. coli* in which mutually inhibitory operons govern the expression of two genes required in two alternative environments; cells reliably switched to the fit state following environmental changes. Stochastic mathematical modeling illustrated the role of gene expression noise in the fitness-induced phenotypic selection in this system. While this phenotypic selection is not as efficient as gene regulation through signal transduction, it represents a simple and generic mechanism for adaptive responses that we term as emergent gene expression (EGE) in this study.

Going beyond bistable systems, Tsuru et al. (20) provided evidence for EGE for a monostable expression of a fitness-conferring gene, using a strain with the hisC gene under the control of a synthetic promoter and under histidine starvation. This response is similar to the “classical” bet-hedging strategy as it relies on cell-to-cell phenotypic variation within the population, but it differs in that it does not require underlying multistability. Moreover, this hypothesis suggests that individual cells within a clonal population (a population characterized by very little or no genetic diversity) could potentially confer a selective advantage over other cells, under a given stress, in a graded manner. The “fitter” cells would display higher growth rates (21), and as a result perpetuate their gene expression pattern in successive generations through epigenetic inheritance (9). Importantly, this leads to a population-wide shift in phenotype. Recent lineage tracing studies have indeed provided evidence for selection of cells based on their phenotypic states (22,23). The advantageous gene may be expressed to a higher degree with every generation through growth-related positive feedback which leads to a graded response to stress over time, where expression patterns shift to favor the advantageous gene (24). Indeed, global positive feedbacks between cellular growth and expression of fitness inducing genes (25) can produce a nontrivial causal relationship between single cell phenotypic state and cell growth (26, 27). EGE could provide an explanation for microbial survival over time and prolonged stress exposure where other modes of survival do not (28). A mathematical model by Mora and Walczak (29) has paved the way for understanding this behavior, where it was shown that a stochastic gene expression model in the presence of stress could cause a unimodal shift to the right (i.e. caused an increase) in the distribution of a fitness-conferring gene. A similar unimodal shift was achieved in a study by Lasri et al. (30), who developed a stochastic gene expression model of O-6-alkylguanine DNA alkyltransferase (MGMT) coupled with cell death in response to temozolomide treatment in glioblastoma cells. Another recent modeling study has taken a phenomenological approach to understanding how tolerance of antibiotics emerges (31), allowing for analytical progress. In a recent synthetic biology study, Camellato et al. (32) engineered a set of gene regulatory networks in the eukaryotic model organism *S. cerevisiae* to control a homologue of the human multidrug resistance gene *MDR1*. They observed that coherent feedforward and positive feedback motifs enable rapid and self-sustained activation of gene expression and enhance cell survival in the presence of cytotoxic drugs.

Due to its nonpermanent nature, EGE behavior is difficult to isolate and characterize. Here, we apply synthetic biology approaches and generate quantitative data to guide mathematical modeling in search of conditions underpinning this EGE behavior. We report a novel dose-dependent relationship of fitness-conferring gene expression in response to increasing selection pressure, which is transient and is based exclusively on phenotypic selection. We, thus propose a new concept in understanding cell population-level antibiotic resistance, which is distinct from—and complements—well-documented survival strategies such as persistence and heteroresistance (33, 34).

## Materials and Methods

### Cell growth

All MK01 *E. coli* strains were cultured in lysogeny broth (LB) and, when indicated, 0.005% (w/v) L-(+)-arabinose (Sigma; stock concentration 5% (w/v) in water) and chloramphenicol (Cm) (Sigma; stock concentration 50 mg/ml in ethanol) of ampicillin (Amp; Sigma; stock concentration 100 mg/ml in water), which were stored at −20°C and added to the medium at the beginning of each experiment.  Histidine was purchased as a 100-mM solution from Sigma. OSU11 and OSU12 *E. coli* strains were a gift from Saburo Tsuru, and were cultured in M63 minimal media and supplemented with histidine as described previously (20). Cm, ampicillin, and histidine were diluted so that 2 µl of an intermediate concentration was added to 148 µl of cells in growth media per well in 96-well plates.

All experiments were inoculated from 5 ml overnight cultures grown in LB without antibiotics at a starting Absorbance_600_ (A_600_) of 0.01 as measured on the Tecan F200 PRO microplate reader. For experiments in OSU11 and OSU12 *E. coli* strains, the overnight culture in LB was inoculated into M63 media supplemented with histidine. The microplate reader format was used for all experiments; cells were grown at 37°C with orbital shaking at 335.8 RPM with an amplitude of 1.5 mm.  For the Rounds experiment, 2 µl of each culture, grown for 12 h, was transferred to 148 µl of fresh LB media (± arabinose and Cm as indicated) to generate an exact replica of the parent plate with the diluted cultures, which were then growth again under the same conditions for 12 h.

### Cassette and strain construction

The *catI* gene was cloned from pTKIP-cat which was a gift from Edward Cox and Thomas Kuhlman (Addgene plasmid # 41065; http://n2t.net/addgene:41065; RRID:Addgene 41065).  Gibson assembly (NEB) was used for all cloning steps, and all constructs were transformed into Top10 *E. coli* (Invitrogen).  The Biobrick part B0034 RBS-*gfp*-Gly4Ser-*cat* construct was inserted downstream of the AraC-pBad cassette in the pCola vector; pCola and GFP were obtained from Schaerli et al. (35). The *bla* gene was cloned from pUC18 which was a gift from Joachim Messing (Addgene plasmid # 50004; http://n2t.net/addgene:50004; RRID:addgene 50004), and inserted to replace the *cat* gene downstream of *gfp* in the pBad-inducible expression cassette described above. Lox sites were inserted to flank a kanamycin resistance gene which was then cloned downstream of the GFP-CAT cassette to aid in selection of genomic integrations.  For constitutive expression, the AraC gene and pBad promoter were replaced by the Biobrick promoter J23100 upstream of the GFP-CAT-lox-kan-lox cassette in the pCola vector. Site directed mutagenesis was performed to generate the *cat-*T_172_A and H_193_A and *bla*-L_74_N mutants within the expression cassettes.  All expression cassette sequences used in this study are reported in Supplementary Material Appendix, Figure S24. A set of plasmids for the main constructs, along with maps and sequences, were deposited in Addgene (IDs are listed in https://www.addgene.org/Mark_Isalan/).

The expression cassettes containing the *cat* gene were integrated into the *intC* locus of the *E. coli* strain MK01 (genotype: F-, Δ(araD-araB)567, ΔlacZ4787(::rrnB-3), λ-, Δ(araH-araF)570(::FRT), ΔaraEp- 532::FRT, φPcp8-araE535, rph-1, Δ(rhaD-rhaB)568, hsdR514, ΔlacI) (36).  The strain was modified to decrease biofilm formation by knocking out the *flu* and *fim* genes.  Briefly, MK01 cells were transformed with the pRed/ET expression plasmid (Gene Bridges kit K006).  Transformants were grown up and recombinase expression was induced as described previously (37). A kanamycin resistance cassette flanked by lox sites was amplified using primers containing sequences homologous to the 5’ and 3’ regions of the *flu* gene and electroporated into the recombinase expressing MK01 cells.  Recombinants were selected on LB-agar containing 15 µg/ml kanamycin (Sigma).  Successful integration was confirmed via amplification and sequencing of the *flu* locus.  The kanamycin resistance cassette was removed by transforming the cells with *Cre* recombinase (Gene Bridges, 706-Cre) according to the manufacturer’s instructions.  This sequence was repeated in order to remove the *fim* locus, and subsequently, to introduce the various CAT-GFP expression cassettes into the *intC* locus.  Genomic integration and sequencing verification primers used in this study are reported in Table S1 (Supplementary Material). All *intC* locus integrations were sequenced to ensure correct integration. Plasmids containing the expression cassettes containing the WT and mutant *bla* gene were transformed into Top10 cells (Invitrogen) and treated with various Amp concentrations as described above.

### Fluorescence and absorbance measurements

All experiments where GFP and RFP fluorescence and Absorbance_600_ (A_600_) were measured were performed in 96-well PS, flat bottom, µClear, black plates (Greiner Bio-One), with *n*= 3 technical replicates per treatment, and *n* = 3 biological replicates unless stated otherwise.  GFP fluorescence was measured at ex485nm/em535nm and a gain value of 25.  Measurements were taken at 15 min intervals.

### Flow cytometry

MK01 cells carrying the *gfp-cat-T_172_A* genomic integration cassette were induced with 0.005% arabinose and treated with 0 and 5 µg/ml Cm, and grown using the microplate reader with GFP and A_600_ measurements performed as above.  At 12 h, 4% paraformaldehyde (Sigma) in PBS was added to each well to a final concentration of 2%, and pipetted up and down to mix.  Cells were stored at 4°C in the dark for 1 to 3 days.  Flow cytometry was performed on a BD Fortessa Analyzer (BD Biosciences) and sample data was analyzed using FlowJo (v10) Software.

### RT-qPCR

MK01 cells carrying the *gfp-cat-T_172_A* genomic integration cassette were induced with 0.005% arabinose and treated with 0 and 5 µg/ml Cm and grown using the microplate reader with GFP and A_600_ measurements performed as above with *n*= 4 technical replicates. Cells were harvested at 1 h time intervals for 11 h.  Briefly, 600 µl of culture of both 0 and 5 µg/ml Cm treatments was removed from the plate, added to 1,200 µl of RNAprotect (Qiagen) in 2 ml Eppendorf tubes, and processed according to the manufacturer’s protocol.  All samples were stored at −80°C until the end of the time course.  RNA was extracted using the RNeasy mini kit (Qiagen). The extracted RNA was treated with TURBO DNA-*free* Kit (Invitrogen) and cDNA was generated using the SuperScript IV First-Strand Synthesis System (Invitrogen). The LightCycler 480 SYBR Green I Master kit (Roche) was used as the qPCR master mix, and the experiments were performed on the Roche 480 LightCycler Instrument II. Housekeeping genes used in this study include *idnT, hcaT*, and *cysG* (38), and were used to quantify *gfp* mRNA expression at each time point. The delta–delta Ct method was used to determine differences in gene expression, with significance determined using the unpaired Student’s t test.  The mean Ct value of the housekeeping genes was also used to normalize Ct values of the control genes *rpoD, rpoH, rpoE, rpoN, acrB, pntB, oppA*, and *cyoC*. Primer sequences used for pPCR amplification are reported in Table S2 (Supplementary Material).

### Mass spectrometry

A volume of 100 µl of culture medium was mixed with 100 µl of a solution containing a mixture of acetonitrile, methanol and water (40:40:20, v/v/v). After centrifugation at 17,000 × *g*, at 4°C, for 10 mins, 100 µl of the supernatant was mixed 100 µl of a solution of acetonitrile containing 0.2% acetic acid. After vortexing and centrifugation at 17,000 × *g*, at 4°C, for 10 mins, 100 µl of the supernatant was loaded into LC-MS vials prior to analysis.

Aqueous normal phase liquid chromatography was performed using an Agilent 1290 Infinity II LC system equipped with binary pump, temperature-controlled autosampler (set at 4°C) and temperature-controlled column compartment (set at 25°C), containing a Cogent Diamond Hydride Type C silica column (150 mm × 2.1 mm; dead volume 315 µl). A flowrate of 0.4 ml/min was used. Elution of polar metabolites was carried out using solvent A (0.2% acetic acid in deionized water (Resistivity ∼18 MW cm), and solvent B (acetonitrile and 0.2% acetic acid). Mass spectrometry was carried out using an Agilent Accurate Mass 6545 QTOF apparatus. Nozzle Voltage and fragmentor voltages were set at 2,000 V and 100 V, respectively. The nebulizer pressure was set at 50 psig and the nitrogen drying gas flow rate was set at 5 l/min. The drying gas temperature was maintained at 300°C. Data were collected in the centroid mode in the 4 GHz (extended dynamic range) mode (39), and the values were normalized to the starting concentration measurement at 0 h.

### Mathematical modeling

We generated agent-based models of the genetic networks that includes stochastic simulation of gene expression inside growing and dividing cells to capture EGE. In our simulations, agents are single cells that are growing and dividing and inside each cell there are biochemical reactions that take place. A single cell’s growth rate was then coupled to stochastic expression of a fitness inducing gene inside that cell. In order to simulate our models we used a mixture of the stochastic simulation algorithm to capture gene expression dynamics and analytical solutions of exponential or logistic growth models to capture the cell growth dynamics. Upon division, we assumed that the the cell’s mRNA and protein contents were binomially distributed between two daughter cells. To manage the computational complexity, we simulated a fixed number of cells where upon any cell division, the new offspring replaces one of the old cells in the population at random. Specifically, we simulated the evolution of the state-matrix *M_i, j_*(*t*), a matrix containing the quantities of the molecular species *j* in cell *i* at time *t* in the model. Another matrix, *P_i, j_*(*t*) was used to store the propensities of the reactions in the system. A third matrix *K_i, j_*(*t*) was used to represent the state-change matrix, which stores the changes in the number of the different molecular species at each time step and was used to update the state-matrix *M_i, j_*(*t*). This matrix *K_i, j_*(*t*) was computed using the propensities from *P_i, j_*(*t*) and updated as per the stochastic simulation algorithm. This was repeated until the volume of the cell reaches its final volume and division occurs. We used Approximate Bayesian Computation (ABC) to fit the models to data (40,41). The full details of the mathematical modeling are found in Supplementary Material Appendix 2. All the modeling was implemented in the Julia programming language.

## Results

**Fig. 1.**
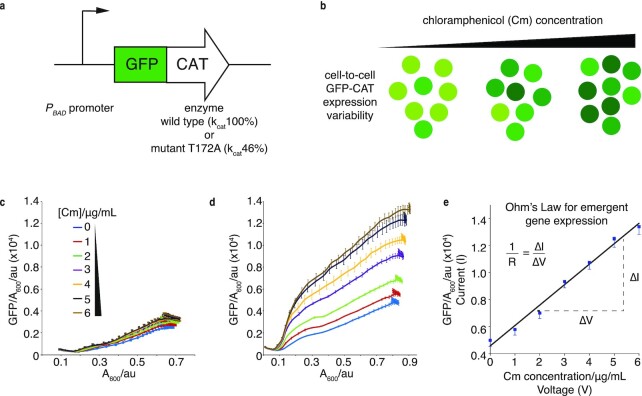
EGE increases linearly with rising antibiotic concentration in analogy to Ohm’s law. (a) Schematic of GFP-CAT expression cassettes used in this study with inducible (*P_BAD_*) promoter driving expression of WT or mutant *cat_T172A_* (relative acetylation efficiencies of k_cat_100% and k_cat_ 46%, respectively). (b) Schematic of EGE for antibiotic resistance. GFP-CAT concentration per cell is indicated by a range of light to dark green; three separate populations are shown where higher *gfp-cat* expression is spontaneously selected in the presence of increasing Cm concentration, to increase cell fitness. (c) and (d) Emergent *gfp-cat* expression with a weakly induced *P_BAD_* promoter (0.005% arabinose). Graphs show GFP/A_600_ per well of populations expressing genome-integrated wt *gfp-cat* (c) and mutant *gfp-cat_T172A_* (d). These were treated with 0, 1, 2, 3, 4, 5, or 6 µg/ml Cm to induce EGE, monitored for 12 h and compared at corresponding Abs_600_; *n* = 3 biological replicates. (e) Ohm’s law analogy for EGE, where GFP-CAT expression is analogous to current (I), Cm concentration to voltage (V), and the slope of the linear relationship between these, representing cellular propensity to increase EGE, is analogous to conductance (1/R). The data points are the maximal EGE values in (d).

To isolate EGE, we applied a selection pressure, using an antibiotic challenge with the protein synthesis inhibitor Cm, and analyzed phenotypic selection output based on changing expression levels of chloramphenicol acetyl transferase (CAT). We integrated wild type (WT) and mutant versions of *gfp–cat* fusion constructs, driven by an arabinose-inducible pBAD promoter, into the *E. coli* genome (Fig. 1a). The pBAD–AraC induction system is well-characterized and frequently used in designing synthetic gene expression cassettes (4). The *E. coli* strain used in this study has been modified to carry a deletion in arabinose metabolizing genes and has been engineered to allow for a graded, rather than all-or-nothing, response to arabinose induction (4). Such a tightly controlled system was essential for providing a stable and predictable level of gfp-cat expression in the absence of Cm. This, in turn, allowed for clear distinction in expression levels upon a graded Cm challenge. In addition to the arabinose-inducible pBAD promoter, we also studied a constitutive promoter system. Constitutive promoters employed in synthetic biology studies are well-characterized in *E. coli* and are not known to be controlled by other elements of the native cellular machinery. In this study, we attempted to uncouple transcription of the engineered cassettes from any native cellular machinery or from promoters associated with expression of the genes under investigation so that we could better predict the expression of this gene product. We employed a synthetic constitutive promoter of a known relative strength to generate a stable level of the gfp-cat cassette expression in the absence of any native inducer or Cm. Thus, similarly to the inducible case of pBAD-mediated gene expression, an increase in gfp-cat expression in the presence of Cm could be associated directly to the pressure applied by addition of the antibiotic. We expected that as Cm concentration increased, cells expressing higher levels of GFP-CAT would be more fit; consequently, *cat* expression and GFP fluorescence within the entire population would rise in a dose-dependent manner (Fig. 1b). Additionally, we reasoned that strains expressing less-active mutant versions of CAT would require higher expression levels than WT, in order to acetylate and neutralize equal amounts of antibiotic.

We challenged populations of cells weakly induced to express either the WT *gfp-cat* cassette or the mutant (T172A, ∼46% of k_cat_ WT activity (42)), with increasing concentrations of Cm, and collected growth and GFP fluorescence time-series measurements. As expected, this resulted in slight upregulation of WT *gfp-cat* expression (Fig. 1c), and in strong, nontransient, Cm-dependent increases in mutant GFP-CAT_T172A_ production, well-above the amount induced by arabinose alone (Fig. 1d). Strikingly, in all cases the increase in fitness-conferring gene (*cat*) expression was linearly correlated to rising selection pressure (Cm concentration) (Fig. 1e).

We find it helpful to understand this observation using an analogy taken from electrical conductivity. In Ohm’s law, current (I) is proportional to voltage (V, electrical pressure), with resistance (R) being the opposition to electron flow. Here, by analogy, the selection pressure (Cm concentration, “voltage”) drives a proportional increase in *gfp-cat* expression (“current”—for simplicity we use the peak expression but found similar results using the mean or median expression) with the metabolic and resource cost of gene expression accounting for the “resistance”, while the slope of the graph (1/R, Fig. 1e) gives the “conductance” of the system, or the propensity of the cell to increase EGE per unit selection pressure.

Next, we applied a series of controls to test the EGE hypothesis. These included addition of a constitutively expressed *cat* gene to the *gfp-cat*_T172A_-expressing strain, thus relieving the selection pressure and obviating the need for EGE. (Figure S1, Supplementary Material). Furthermore, *gfp* expression did not increase upon Cm treatment in a strain encoding a functionally inactive *gfp-cat_H193A_* mutant, suggesting that EGE selects only those cells which contain increased amounts of a functional fitness-conferring protein, and that Cm does not directly activate the promoter (Figure S1, Supplementary Material). We also found this phenotypic selection effect to be reversible and reproducible over several short rounds of antibiotic challenge and washout, reflecting the inherent flexibility of the mechanism (Figure S2, Supplementary Material). In addition, RT-qPCR analysis showed that *gfp-cat* transcripts were specifically upregulated upon Cm treatment, while expression of housekeeping genes, sigma factors, and other antibiotic treatment response genes remained constant (Figure S3, Supplementary Material). This suggests that phenotypic selection based on Cm-induced EGE is specific to the *gfp-cat* gene, that the level of upregulation is reversible and that it is related to the per-molecule activity of the fitness-conferring enzyme.

To understand the mechanisms underlying EGE, we established minimal requirements necessary to recapitulate this behavior in silico. We constructed agent-based models that included growth and division of cells exhibiting stochastic gene expression of a fitness-conferring gene (40,43, 44). Previously several studies have indicated possible global links from cell growth rate to both transcription and translation (25,45–47). Therefore, we performed a comparison of 10 toy models exploring a range of assumptions for gene expression and growth regulation (Table 1). Our toy models contained two genes, a fitness-conferring gene and a reference gene, which we assumed were modeled with the same parameters with the only difference being that the growth rate of the cell was coupled to fitness-conferring gene expression. We assumed cell growth rate was constant in the absence of stress and became more dependent on the fitness-conferring gene as the stress level increased. We simulated cell populations in a chemostat-like setting (where a constant number of cells was tracked) and we performed ABC (41) model selection to find models that can show an increase in the fitness-conferring gene relative to the reference gene. Each of the models possessed different levels of biological detail with some only possessing mRNA and others having mRNA, protein, and known global links between gene expression and cell growth. More details about the reactions of each of the 10 models, parameter inference, and the error form chosen to maximize EGE can be found in Appendix Sections 1.3 and 1.4.

**Table 1.** The upper part of the table lists the different model assumptions made. A tick indicates that the model (for a given column) contains an assumption (given by the row) while a cross indicates that it does not. The lower part (highlighted in gray) summarizes the model output. The first row indicates whether each model is capable of producing a linear relationship between the fitness protein and the selection pressure. The second row shows whether we can observe a unimodal shift to the right (increase in the production) of the fitness protein in response to stress. The next row shows whether an increase in the mean mRNA level is observed in response to selection pressure. Finally, the maximum mean ratio of the fitness protein to the reference protein for each model is displayed in the last row. The only model that captures the observed data (unimodal shift, mRNA increase, and Ohm’s Law) is model 8, which also worked when tested for the regulated case and which we adopt in the main paper.

Model	1	2	3	4	5	6	7	8	9	10
mRNA	✓	✓	✓	✓	✓	✓	✓	✓	✓	✓
Protein	✗	✗	✗	✗	✓	✓	✓	✓	✓	✓
Biased partitioning	✗	✗	✗	✗	✓	✗	✗	✗	✓	✗
Global transcription feedback	✗	✗	✗	✓	✗	✓	✗	✓	✗	✗
Global translation feedback	✗	✗	✗	✗	✗	✗	✓	✓	✗	✗
Constitutive	✓	✓	✓	✗	✓	✓	✓	✓	✓	✗
Regulated	✗	✗	✗	✓	✗	✗	✗	✗	✗	✓
No. of parameters	2	3	3	4	3	4	4	5	4	5
Ohm’s Law	✗	✗	✗	✗	✗	✗	✓	✓	✗	✓
Unimodal shift	✗	✗	✗	✗	✗	✗	✓	✓	✗	✗
mRNA increase	✗	✗	✗	✓	✗	✗	✗	✓	✗	✓
Max ratio	1.0	1.0	1.0	1.0	1.0	1.0	1.75	1.8	1.0	2.1

Models 7, 8, and 10 exhibited EGE (Table 1). Model 7 won in the ABC model selection as it is the model with the least number of parameters that can produce EGE. We found that growth-dependent dilution and a global positive feedback coupling translation rate to cell growth were essential (Table 1). There is evidence for this coupling in the literature (47, 48). Such positive feedbacks can extend the lifetime of protein fluctuations beyond the dilution time set by the cell cycle (49, 50). The candidate models also explicitly required both transcription and translation components to be modeled to produce EGE, as removing the mRNA variable did not produce sufficient levels of noise for phenotypic selection to act upon (Supplementary Material Appendix, Figures S6–S9). We note that a saturating rather than linear dependence between the translation rate and growth rate (as in (47)) was sufficient to produce EGE (see Supplementary Material Appendix, Figure S10).

**Fig. 2.**
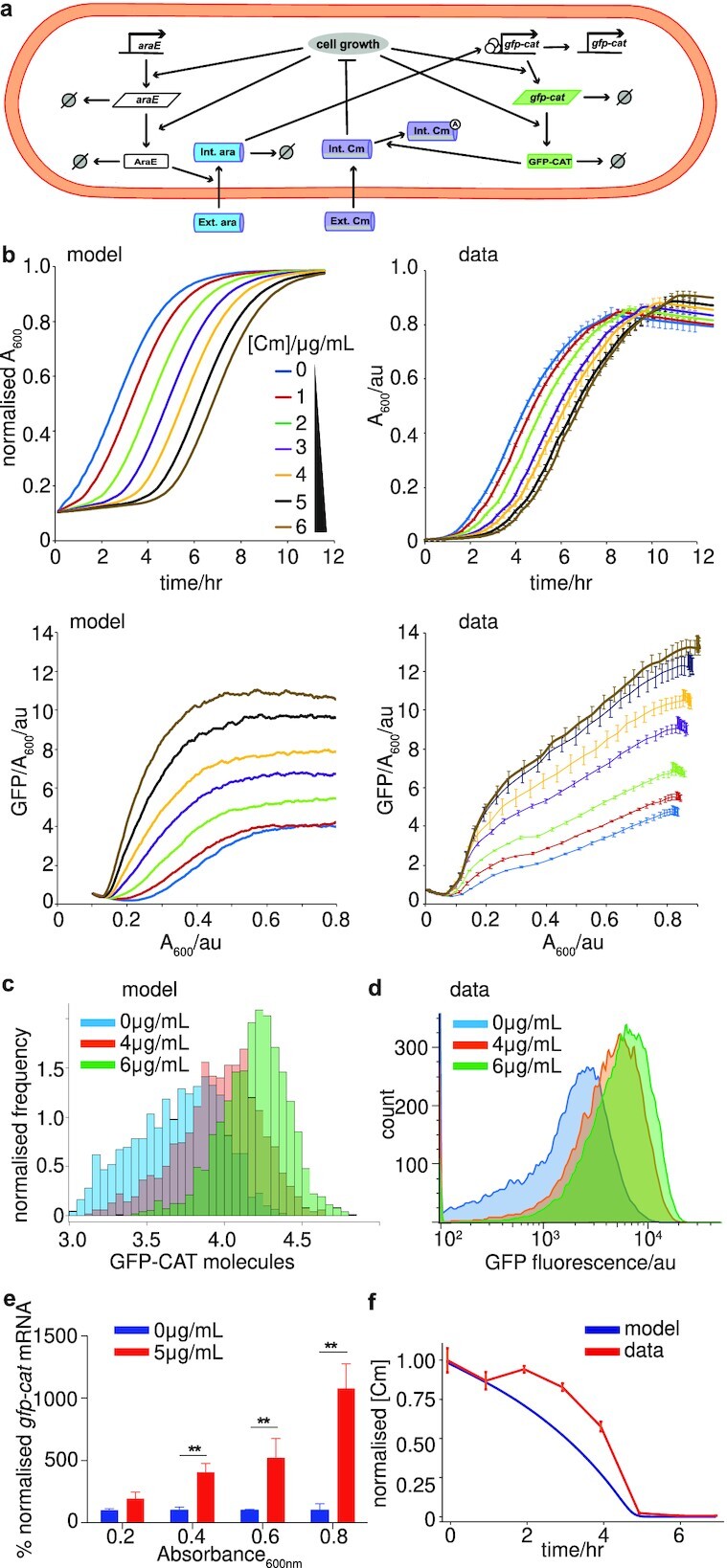
Experimental verification of the inducible promoter computational emergence model. (a) Schematic for mathematical modeling of the inducible promoter system for a single cell. The reactions capture *gfp-cat* expression and degradation, *araE* expression and degradation, arabinose import, degradation and activation of the *P_BAD_* promoter, as well as Cm import and acetylation by GFP-CAT. Intracellular dynamics were coupled to a second model scale, which captured cell division, partitioning and logistic growth of the cell population (more details in Appendix 2 and Supplementary Material Appendix, Figures S11–S14). (b) Comparing model outputs with experimental data for growth (normalized A_600_ data) and GFP-CAT molecules per cell (GFP/A_600_), for increasing Cm concentrations, over 12 h. Best fit parameters were taken from ABC parameter inference and initial conditions (initial number of cells and GFP expression levels) were taken from the experimental data displayed in right hand panels. mRNA levels were assumed to be zero initially. (c) and (d) Comparing model outputs with experimental data for population distributions of EGE. GFP-CAT molecule distributions are shown for increasing Cm concentrations. The *x*-axis in (c) is displayed on a log10 scale and the *y*-axis is scaled such that the total area of the histograms sum to 1. The flow cytometry analysis (d) of the genome-integrated *gfp-cat_T172A_* mutant matches the model prediction (c). Cells in (d) were weakly induced with 0.005% arabinose, treated with 0, 4, or 6 µg/ml Cm for 12 h, and fixed in 2% paraformaldehyde; *n* = 3 biological replicates. (e) RT-qPCR assay of *gfp-cat_T172A_* mRNA expression in populations induced with 0.005% arabinose, treated with 0 (blue) or 5 (red) µg/ml Cm, and harvested at A_600_ of 0.2, 0.4, 0.6, and 0.8 (Mean ± SEM; *n*= 3 biological replicates). Asterisks represent *P-*values: ** = *P* < 0.01. (f) Cm concentration in cultures of the genome-integrated *gfp-cat_T172A_* mutant strain, measured by LC-MS at 1-h intervals (orange). This is compared with the predicted Cm concentration from simulation of the refined inducible promoter model (Supplementary Material Appendix, Figure S23). Both time series are normalized to their maximal values.

Establishing the minimal requirements for the EGE using our toy models in a chemostat setting, we attempted to quantitatively explain our microplate data using a specific model of the pBAD inducible GFP-CAT system (Fig. 1a). Given Model 8 was the only toy model that showed robust EGE coupled with a uni-modal shift in gene expression at the protein level as well as mRNA upregulation (which we observed experimentally, see Fig. 2e), we decided to use toy Model 8 as the basis of our model of the pBAD inducible GFP-cat system (Fig. 2a). We also modeled arabinose and Cm explicitly in our model with Cm passively diffusing into cells where it hinders cell growth and arabinose import being dependent on AraE expression. We also assumed that the level of intracellular arabinose determined the activity level of the pBAD promoter and that the pBAD promoter becomes inactive at a constant rate. We also assumed that GFP-CAT was able to acetylate intracellular Cm and that acetylated chlorampenicol does not impact cell growth. To infer parameters for this system, we again used ABC (41) but this time in conjunction with gene expression time series data and growth kinetics data from our microplate experiments (Fig. 2a and b; Supplementary Material Video 1). We note that there is a slight discrepancy between the presented growth kinetics data and that which was used for fitting the model using ABC. To avoid the simulations beginning from zero or close to zero cells, we did not remove the constant background OD from the data used for the fitting. We also did not model cell death in this particular model, so we ignored the observed decrease in OD levels during the stationary phase part of growth.

Importantly (as was the case for Model 8) without further fitting, the model was able to predict a Cm-dependent unimodal increase in CAT expression across the entire distribution of the population (Fig. 2c), which we validated using flow cytometry (Fig. 2d). The model similarly produced experimentally observed increasing *gfp-cat* mRNA levels in the presence of increasing Cm concentration (Fig. 2e; Supplementary Material Figure S3). We also used mass spectrometry to measure the time–course of external antibiotic depletion and this was also captured qualitatively by our model without further fitting (Fig. 2f; see Appendix Section 1.6.3 for a revised model that captures mass spectrometry data more accurately). We also observed the reversibility of EGE upon removal of Cm after multiple washouts (Figure S2, Supplementary Material), but we noticed a plasticity for the observed EGE, suggesting metastability of the pBAD promoter. In line with these observations, our model predicts the timescale of this increased promoter activity is dependent on the promoter deactivation rate as shown in simulated washout experiments (where we remove Cm from the system and re-run numerical simulations iteratively using final states as initial states) shown in Supplementary Material Appendix, Figures S19 and S20. In short, the mechanism of the model of the inducible pBAD system can be understood as follows. The global feedbacks between gene expression and growth can lead to more arabinose import during log phase of growth and the subsequent activation of the pBAD promoter acts to push the system to a metastable state of higher fitness-conferring gene expression.

**Fig. 3.**
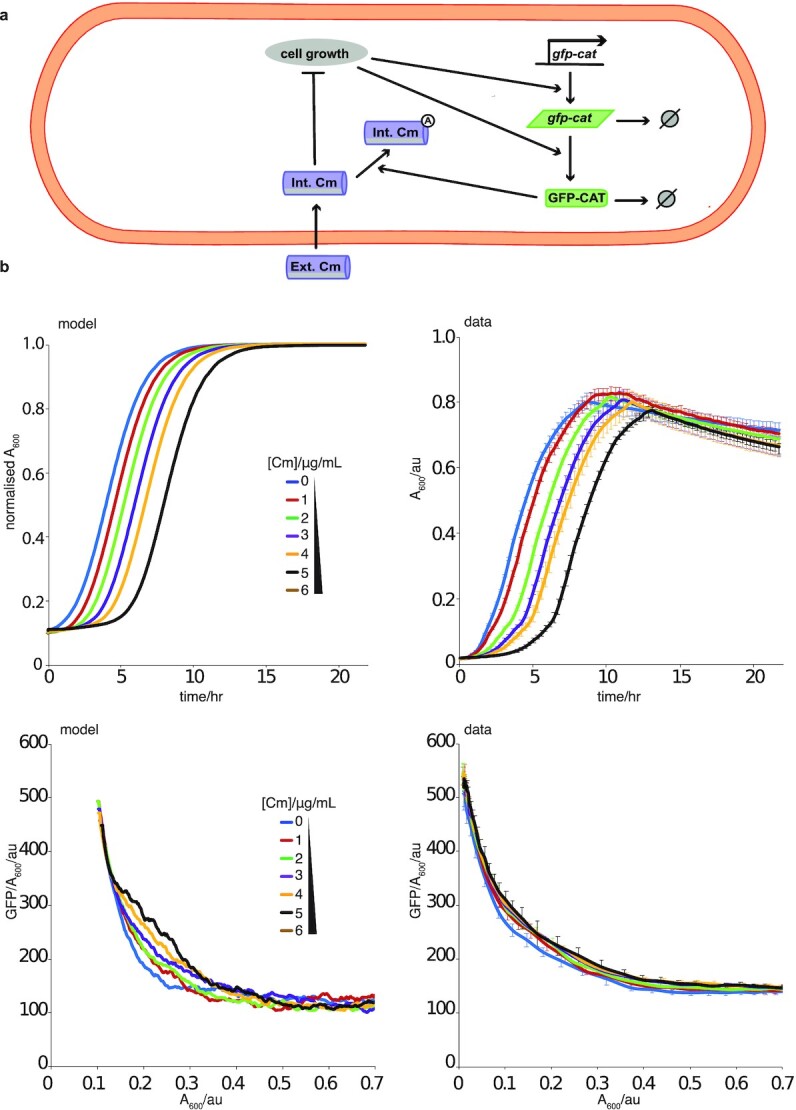
Experimental verification of the constitutive expression computational emergence model. (a) Schematic for mathematical modeling of the constitutive promoter system for a single cell. The reactions capture *gfp-cat* expression and degradation, as well as Cm import and acetylation by GFP-CAT. Intracellular dynamics were coupled to a second model scale, which captured cell division, partitioning, and logistic growth of the cell population (more details in Appendix 2 and Supplementary Material Appendix, Figures S11–S14). (b) Comparing model outputs with experimental data (promoter BBaJ23100 with mutant T_172_A GFP-CAT). Graphs show mean (± SDM) GFP expression (GFP/A_600_ per well) in populations constitutively expressing genome-integrated mutant *gfp-cat_*T*172*A*_*. These were treated with 0 to 6 µg/ml Cm, to induce EGE, and monitored for 22 h; *n*= 3 biological replicates. Time series showing total number of cells from simulation of a constitutive promoter model normalized by carrying capacity (upper left panel), corresponding experimental mean (± SDM) GFP expression (GFP/A_600_ per well) in populations constitutively expressing genome-integrated mutant *gfp-cat_*T*172*A*_*. (upper right panel), mean number of GFP-CAT molecules per cell from simulation of the constitutive promoter model (lower left panel) and corresponding experimental mean *gfp* expression data (lower right panel), for cells treated with 0 to 6 µg/ml Cm, for a time period of 22 h. Best fit parameters were used from ABC parameter inference and initial conditions (initial number of cells and *gfp* expression levels) are takenwere taken from data displayed in right hand side panels. mRNA levels were assumed to be zero initially.

Next, we observed and modeled EGE behavior in the context of constitutive expression of the *gfp-cat_T172A_* cassette, where we again saw a dose-dependent unimodal increase in *gfp-cat_T172A_* expression (Fig. 3; Figure S4, Supplementary Material; Supplementary Material Video 2). Mathematical modeling of this constitutive expression model was able to reproduce our data (Fig. 3—again we rescaled the growth kinetics data as in the pBAD inducible system). In this case, the EGE observed was more transient than in the inducible pBAD system case. We understood this difference arose from the fact that EGE required growth and growth feedbacks on gene expression. Hence, by the time the cells entered the stationary phase it was no longer possible to observe, as the growth rate slows it becomes weaker (see Figure S4, Supplementary Material). This is why we see the largest effect at the log-phase of growth. In contrast, the pBAD inducible promoter system yielded less transient levels of EGE in the form of increased promoter activity.

**Fig. 4.**
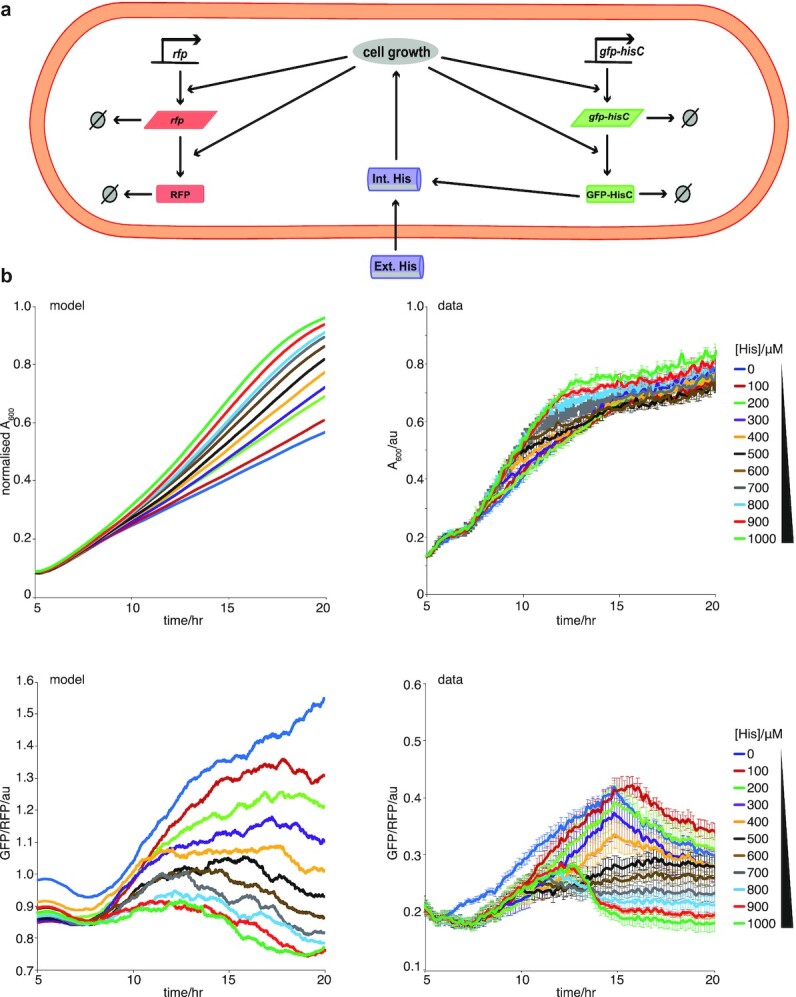
Experimental verification of inducible expression model of EGE of *gfp*-*hisC* in *E. coli OSU12-hisC* grown in decreasing concentrations of histidine in the growth medium. (a) Schematic for mathematical modeling of the histidine depletion system for a single cell. The reactions capture *gfp-hisC* and *rfp* expression and degradation, as well as histidine (His) import. Intracellular dynamics were coupled to a second model scale, which captured cell division, partitioning, and logistic growth of the cell population (more details in Appendix 2 and Supplementary Material Appendix, Figure S22). (b) Comparing model outputs with experimental data for growth (normalized A_600_ data) and GFP-HISC molecules per cell (GFP/A_600_), for decreasing His concentrations, over 20 h. Time series showing total number of cells from simulations of an inducible promoter model normalized by carrying capacity (upper left panel) and corresponding experimental mean A_600_ data (upper right panel) for 11 histidine conditions ranging from 0 to 1,000 µM for a time period of 20 h. Lower plots show the corresponding time series of *gfp* expression normalized to *rfp* expression for the same conditions with the left plot showing the model output and the right plot showing the experimental mean. Best fit parameter were used from ABC parameter inference and initial conditions (initial number of cells, *gfp* expression, and *rfp* expression levels) were taken from data displayed in right hand side panels. mRNA levels were assumed to be zero initially.

To determine whether our observation apply to other biological systems, we were able to isolate and characterize dose-dependent EGE within an entirely unrelated fitness model system previously reported by Tsuru et al. (20). Here, antibiotic exposure was replaced by histidine auxotrophy, which was relieved by expression and upregulation of a histidine biosynthesis pathway gene. Deletion of the native histidinol-phosphate aminotransferase *hisC*, and subsequent rewiring of the strain to encode a monostable *hisC-gfp* circuit, allowed for the uncoupling of *hisC* expression from its native operon. This resulted in a fully synthetic and tunable expression system. We applied a range of selection pressures by gradually reducing the availability of histidine in the medium; we found a corresponding concentration-dependent stochastic upregulation of the *hisC* gene, again demonstrating “gene expression according to need” (Fig. 4b). In order to model this system we again used the toy Model 8 as the basis and for this system we included an added control of a reference gene expressed at the same rate as hisC-gfp (Fig. 4a). We note that in this case, in order to use the same parameters for the reference gene and hisC-gfp gene, we had to rescale the gene expression data so that the ratio of the two genes was 1 at time 0 for 0 histidine (and we also normalized the growth data as in the other systems). We also modeled an external pool of histidine, which we assumed diffused into the cells and could vary at the beginning of our numerical experiments. Furthermore, we assumed that cell growth depended on the level of internal histidine in a saturating manner. By coupling these experimental results to our theoretical framework, again using ABC, we were able to model this behavior. This system also displayed dose-dependent EGE, with less histidine supplied leading to a larger bias in the fitness protein (hisC-gfp) compared to the reference protein (RFP).

**Fig. 5.**
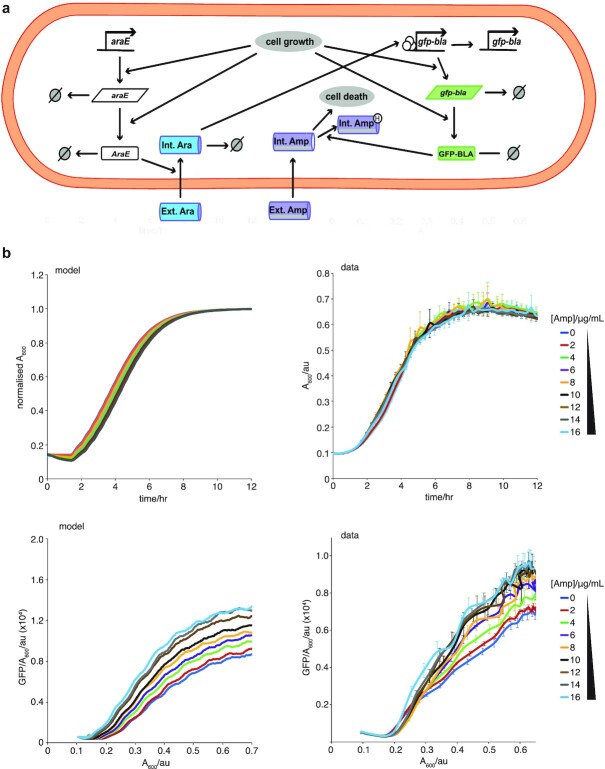
Experimental verification of the ampicillin computational emergence model. (a) Schematic for mathematical modeling of the ampicillin system for a single cell. The reactions capture *gfp-bla* expression and degradation, as well as ampicillin (Amp) import and cleavage by GFP-BLA. Intracellular dynamics were coupled to a second model scale, which captured cell division, partitioning and logistic growth of the cell population (more details in Appendix 2 and Supplementary Material Appendix, Figure S23). (b) Comparing model outputs with experimental data for growth kinetics Abs_600_ (upper panels) and GFP fluorescence per well of populations (lower panels) expressing plasmid-encoded mutant *gfp-bla74N* and treated with 0, 2, 4, 6, 8, 10, 12, 14, and 16 µg/ml Amp; *n* = 3 biological replicates. Best fit parameters were used from ABC parameter inference and initial conditions (initial number of cells and *gfp* expression levels) were taken from data displayed in top panels of (b). mRNA levels were assumed to be zero initially.

Lastly, using our approach of varying fitness-conferring gene product activity, we identified dose dependent EGE in a bactericidal antibiotic resistance context. Here, cells expressing the wild-type or mutant (L74N) (51) beta-lactamase *(bla*) gene from a plasmid template were treated with increasing amounts of ampicillin. In line with our findings for the Cm resistance framework, expression of WT *bla* increased slightly with corresponding increase in Amp concentration (Figure S5, Supplementary Material), while expression of the mutant *bla*_L74N_ was more pronounced at much lower concentrations of the antibiotic (Fig. 5b). The model we developed for this system was similar to the one we developed for the pBAD inducible GFP-CAT system. Through application of ABC, we were able to parameterize our model and find good agreement between the model and data. The main difference between the chlorampenicol and ampicillin models was that we assumed intracellular ampicillin induced cell death with some probability and that cleaved ampicillin no longer could induce cell death. Therefore, cells rich in GFP-BLA were less likely to die and therefore became more represented in the cell populations as ampicillin was administered.

**Fig. 6.**
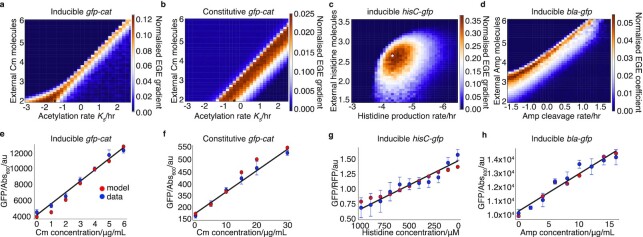
Bands and islands of maximal EGE, in antibiotic resistance and histidine auxotrophy, in parameter space. Heatmaps showing the effect of varying acetylation rate and Cm dosage on a normalized EGE gradient of *gfp-cat*, for (a) inducible (see Supplementary Material Appendix, Figures S15–S17) and (b) constitutive promoter models (see Supplementary Material Appendix, Figures S11–S14). A total of six different simulations were computed per model, corresponding to cells being treated with Cm ((a): 0C, 1C, 2C, 3C, 4C, 5C, or 6C; (b): 0C, 5C, 10C, 15C, 20C, or 30C; where C is the number of external Cm molecules). (c) Heatmap showing effect of varying histidinol-phosphate aminotransferase activity on a normalized EGE gradient of *gfp-hisC*. Linear regression is applied to the HisC-GFP/RFP levels yielded from simulations of model of HisC dynamics (see Fig. 4) generated using decreasing concentrations of histidine from 1,000 to 0 µM for each time point, to obtain the maximum gradient. (d) Heatmap showing the effect of varying the cleavage rate and concentration of ampicillin (Amp). Linear regression is applied to the *gfp-bla_L74N_* expression levels yielded from simulations of model of *bla_L74N_* dynamics (see Fig. 5) generated using concentrations of Amp from 0, 2, 4, 6, 8, 10, 12, 14, and 16 µg/ml for each time point, to obtain the maximum gradient. White asterisks highlight the parameter combinations found to provide the best model fits to the microplate reader data. (e, f, g, and h), Linear regression fits of microplate reader data showing linearity of GFP expression relationship (Ohm’s law) with Cm treatment dosage, for both (e) inducible and (f) constitutive cases of *cat-gfp_T172A_* expression. (e) shows mean (± SDm) GFP expression (GFP/A_600_ per well) in populations treated with 0, 1, 2, 3, 4, 5, or 6 µg/ml Cm at *t* = 12 h (blue points) with linear regression fit overlaid (black solid line). These experimental data were compared with the predicted mean GFP-CAT molecules per cell for inducible promoter model simulations at same time point (red points), (f) is similar, for populations treated with 0, 5, 10, 15, 20, or 30 µg/ml Cm at *t* = 3 h (blue points), compared with model predictions (red points) of the constitutively expressed *gfp-cat_T172A_*. (g) Linear regression fit of *gfp* expression normalized to *rfp* expression in cells grown in minimal media with decreasing concentrations of histidine from 1,000 to 0 µM (red dots) compared with the predicted mean GFP-HisC/RFP molecule production at the respective concentrations. (h) Linear regression fit of inducible *gfp-bla_L74N_* expression (± SDm) in populations treated with 0, 2, 4, 6, 8, 10, 12, 14, and 16 µg/mL Amp at *t* = 12 h (blue points) with linear regression fit overlaid (black solid line). These experimental data were compared with the predicted mean GFP-CAT molecules per cell for inducible promoter model simulations at same time point (red points).

Fitness-induced gene expression effects in antibiotic resistance have been reported previously, however, due to the transiency of this phenomenon, collection of corresponding fine-grained experimental data for model fitting is difficult (23,52, 53). In this study, the critical difference is that we used decreased-activity fitness-conferring gene mutants that enhance EGE to observe and quantify dose-responses. Similarly, using mathematical modeling, we revealed that to observe maximal EGE magnitude, one needs to use intermediate fitness-conferring gene strength (*cat_T172A_*) and intermediate selection pressure (Cm; Fig. 6a). This interplay between fitness and strength of selection resulted in theoretically detectable bands or islands of maximal EGE within the fitness parameter landscape (Fig. 6a). Similar to this case we observed islands of maximal emergence by modulating enzymatic activity *in silico* in the other cases of constitutive promoter (Fig. 6b), HisC system (Fig. 6c) and ampicillin resistance (Fig. 6d). For all these systems, varying selection pressure within an appropriate range yielded a linear Ohm’s-law-like EGE (Fig. 6e–h).

## Discussion

In summary, we report fitness-induced EGE, and corresponding mathematical model banding patterns based on expression strength and fitness-conferring gene activity, for both plasmid and genome integrated expression systems. This is in the case of both bacteriostatic and bactericidal antibiotics, as well as auxotrophy supplementation, indicating the potentially wide-spread nature of this population-level behavior. We were, thus able to build an accurate predictive model of EGE, explaining the associated fitness-conferring gene expression increases in the presence of a selection pressure. Based on these findings, we propose that population-level noise in gene expression ensures the existence of cells with a range of fitness and that higher expression of a fitness-conferring gene results in faster division times in the presence of a corresponding selection pressure. Furthermore, positive feedbacks between gene expression and cell growth produces a memory effect where daughter cells inherit the level of fitness-conferring gene, thus making phenotypic selection and EGE possible.

Our mathematical and experimental results show that EGE is a phenomenon that can be observed with some degree of tuning of the relevant parameters and may play a role in the expression of many fitness-conferring genes. While each of the systems we explored and modeled had their own specific elements (and we refer the reader to the supplemental material for details), each of the systems contained the crucial elements of a growing cell population with stochastic expression of a fitness-conferring gene in single cells leading to faster cell growth and positive feedbacks between gene expression and cell growth. One of the most critical parameters we found was the level of stress or selection pressure. In particular we found (both experimentally and computationally) that if the selection pressure was too great that EGE was not possible. Hence, EGE is a phenomenon that only occurs under specific conditions (for example in the case of the inducible gfp-cat system, for the external chlorampenicol pool fixed to 10^4^ molecules we find EGE for the acetylaton rate in the approximate range 0.2 to 1.0 per hour). It should be noted that even small changes in the expression of highly active fitness-conferring genes, such as those encoding antibiotic resistance enzymes, may be crucially important for cells’ biology even where transient EGE may be difficult to detect with current experimental methods (54). Mathematical modeling reveals these relationships and indeed confirms that an intermediate-activity mutant maximizes EGE and gives a model output that fits remarkably well with the experimental Ohm’s-law-like linear framework of dose dependent gene expression (Fig. 6a–d). This can be observed within inducible and constitutive expression systems and across different fitness-conferring genes (Fig. 6e–h). The generality of our results could be further tested by performing similar experiments using other fitness inducing genes in other bacteria or eukaryotic cells under a range of different selection pressures. In principle, we suspect the same effect could be observed when cancer cells are exposed to a chemotherapeutic stress (30).

The observed Ohm’s-law-like dose-dependent EGE could be viewed as an example of a more general fluctuation–response relationship that has been proposed before (55,56). The quantity of interest here is expression of fitness inducing gene, and the response is stress dose-dependent EGE. According to this fluctuation–response relationship the response (EGE) to a fixed amount of force (stress) should be proportional to the fluctuation in the quantity of interest in the absence of force, which in our case is noise in the uninduced gene expression. Indeed, as shown in the appendix, in our exploration of our toy models this can be observed for models with unimodal EGE (Models 7 and 8; see Figures S7 and S8, Supplementary Material), but not models with bimodal expression (Model 10; see Figure S9, Supplementary Material). So, the origin of the observed EGE Ohm's-law can be traced back to the general fluctuation–response relationship for Gaussian-like distributions (see (55) for a derivation of this result).

We emphasize that our results are based on isogenic cell lines and further work is required to investigate the interplay between EGE, genetic mutations, and evolution. While, our results are of relevance to ecological time-scales and we observed no mutations during our experiments contributing to EGE, future studies could investigate evolutionary consequences by repetitively propagating cultures over many days under the conditions where EGE is expected. We note that, over evolutionary time, higher adaptedness might require genetic mutations and natural selection, and thus EGE behavior might be prevented from general success on evolutionary timescales. Indeed, the effects are magnified by “inferior” genes such as the CAT mutants exemplified here. EGE may, therefore, be more of a general bridge to temporary survival under new conditions, until mutation and natural selection have time to catch up. This is consistent with what is proposed by Dunlop et al. (57) with respect to efflux pumps. Moreover, a recent study showed that phenotypic heterogeneity in bacteria populations could be increased following application of intermediate antiobiotic doses (58). Phenotypic heterogeneity can be further amplified due to genetic mutations (see (59) for a review of this subject). So, the evolution of noisy gene expression, its interplay with EGE and evolutionary adaptation could be promising areas of future research.

Overall, our results indicate that the linear relationship between selection pressure and gene expression relies on phenotypic selection requiring cellular growth and division, growth positive feedbacks, stochasticity, and fitness-conferring gene activity. We hypothesize this kind of need-based gene expression increases population survival in the presence of stresses such as antibiotics or cancer-targeting drugs, which could preclude or precede the necessity for hardwired genetic changes.

## Supplementary Material

pgac069_Supplemental_Files

## Data Availability

All data is included in the manuscript and/or supporting information.
